# Molecular Progress in Research on Fruit Astringency

**DOI:** 10.3390/molecules20011434

**Published:** 2015-01-15

**Authors:** Min He, Henglu Tian, Xiaowen Luo, Xiaohua Qi, Xuehao Chen

**Affiliations:** School of Horticulture and Plant Protection, Yangzhou University, 48 East Wenhui Road, Yangzhou 225009, Jiangsu, China; E-Mails: 18796600467@163.com (M.H.); THL1183528252@163.com (H.T.); 15861323316@163.com (X.L.); xhqi@yzu.edu.cn (X.Q.)

**Keywords:** fruit astringency, tannin, biosynthesis pathway, regulation

## Abstract

Astringency is one of the most important components of fruit oral sensory quality. Astringency mainly comes from tannins and other polyphenolic compounds and causes the drying, roughening and puckering of the mouth epithelia attributed to the interaction between tannins and salivary proteins. There is growing interest in the study of fruit astringency because of the healthy properties of astringent substances found in fruit, including antibacterial, antiviral, anti-inflammatory, antioxidant, anticarcinogenic, antiallergenic, hepatoprotective, vasodilating and antithrombotic activities. This review will focus mainly on the relationship between tannin structure and the astringency sensation as well as the biosynthetic pathways of astringent substances in fruit and their regulatory mechanisms.

## 1. Introduction

Recently, the quality of fruits and vegetables has become increasingly important in people’s daily lives. Fruit quality can generally be divided into the following three components: the first is commercial quality, which includes the fruit’s outer appearance, fruit length and diameter; the second is fruit structural quality, for example, in terms of flesh thickness and cavity size; and the third is fruit sensory quality. Regarding oral sensory quality, there are six oral sensory characteristics of fruit: sourness, sweetness, bitterness, spiciness, aroma and astringency. For many people, the oral sensory properties of fruit have a great impact on the choice, acceptability and consumption of fruit. Sourness in fruits mainly comes from organic acids, such as citric acid, malic acid and tartaric acid as well as small amounts of oxalic acid, succinic acid and salicylic acid. Sucrose, fructose and glucose and their derivatives, such as sugar alcohols, are the main source of carbohydrate-related sweetness in fruit. Some amino acids, amines and other non-carbohydrates can also contribute to a sweet taste. Glycoside substances formed by sugar free radicals and aglycone, linked through glycosidic bonds, such as amygdalin, naringin and neohesperidin, are the cause of bitterness in fruits. The main spicy substances found in pepper fruits mostly consist of capsaicin. Fruit aroma is due to alcohols, esters, aldehydes, ketones and aromatic terpenes as well as other volatile substances [1]. Astringency mainly comes from tannins and other polyphenolic compounds. Astringency is a tactile sensation which has been also described as an oral sensation that causes drying, roughing and puckering of the mouth epithelia [2]. It has been classically attributed to the interaction between tannins and salivary proteins [3], leading to precipitation. Phenolic compounds present in wine, especially tannins, have been widely related to the perception of astringency [4,5,6].

There is growing interest in the study of fruit astringency because of the healthy properties of astringent substances found in fruit including antibacterial, antiviral, anti-inflammatory, antioxidant, anticarcinogenic, antiallergenic, hepatoprotective and vasodilating [7]. This review will focus mainly on the relationship between tannin production and astringency formation as well as the biosynthetic pathways of astringent substances in fruit and their regulatory mechanisms.

## 2. Tannins and the Generation of Fruit Astringency

Tannins are the key factor determining the degree of astringency in fruits. All astringency removal methods base on the reaction of acetaldehyde with soluble tannins forming an insoluble non-astringent compound [8].

### 2.1. Astringency and the Antioxidative Activity of Tannins

Combining tannins with saliva proteins results in precipitation and leads saliva to lose its lubricity, causes tongue epithelial tissues to contract, producing a dry feeling [9]. This process is referred to as convergence or astringency. Studies have shown that a large number of hydrophobic groups in protein molecules form a “hydrophobic pocket” to combine with tannins. Tannins with phenolic hydroxyl groups and hydrophobic groups bind within the “pocket” and combine with each other via hydrogen bonding [10]. The cross linking of tannins and a number of proteins causes protein cohesion, leading to precipitation [11,12]. Therefore, when humans consume a certain amount of tannins, the cross linking with salivary proteins and gastrointestinal dietary proteins results in an abnormality of protein metabolism that may affect the digestibility and utilization of proteins [13].

Tannins are polyphenols and their molecular structure contains a number of phenolic hydroxyl groups, and they can readily react with oxygen radicals. In addition, tannins can release a large amount of hydrogen ions that can combine with free oxygen radicals [14]. Therefore, tannins can resist the senescence of tissues and organs and a variety of aging diseases (cardiovascular disease, aging, cataracts) [15]. Salah *et al.* discovered that the greater the degree of polymerization of tannins, the higher the number of phenolic hydroxyl groups and the stronger the inhibition of free radicals [16]. Okuda *et al.* studied 25 different types of tannins and other relevant compounds, 23 of which demonstrated antioxidant activity to differing degrees, depending on the location and number of phenolic hydroxyl groups [17].

The phenolic hydroxyls in tannins can interact with a variety of metal ions and cause an oxidation-reduction reaction, consequently reducing the metal ions from high valence to low valence and finally condensing them into a red-brown or brown quinone product. Reactions between tannins and metal ions reduce the absorption of calcium and iron by the human body. However, interaction of tannins and calcium outside of tissues can lower blood pressure [18].

### 2.2. The Molecular Structure and Polymerization of Tannins and Their Relationship with the Intensity of Astringency in Fruit

The molecular weight of tannins ranges from 500 to 3000 Daltons [3]. They are amphipathic molecules, containing both hydrophobic aromatic rings and hydrophilic hydroxyl groups, allowing them to bind simultaneously at several sites on the surface of other molecules [19]. In 1920, K. Frendenberg divided tannins into two categories based on structural features: hydrolyzed tannins and condensed tannins. Hydrolysable tannins are heterogeneous multimers. They are relatively low molecular weight polyphenols formed from acids and their derivatives together with glucose or polyhydric alcohols through ester bonds. They are readily hydrolyzed into simple compounds by acids, alkalis and enzymes and can be divided into gallic and ellagic tannins. The former group can be hydrolyzed into gallic acid, and the latter can be hydrolyzed into inverse gallic acid. Condensed tannin, also known as proanthocyanidin, is a polymer (three or more monomers polymerized) composed of hydroxy flavan-3-ol (whose derivative is catechin) and flavan-3 hydroxy-4-diol (whose derivative is a colorless anthocyanin). Proanthocyanidin exhibits a relatively high molecular weight and more stable chemical structure, but it can be condensed into anthocyanin using hot acid.

There are several variables related to tannins that are highly correlated with the perception of astringency, such as their total concentration, average degree of polymerization (aDP) [6], subunit composition and distribution [20]. Because tannins are polymers of flavan-3-ol subunits, they present a wide range of possible molecular weights [21,22]. Tannins vary in size from dimers and trimers up to oligomers with more than 30 subunits [23]. Polymer size is the most discriminatory structural variable affecting the intensity of astringency, which correlates positively with the perception of astringency [24,25,26]. Increased galloylation could be responsible for increased “coarseness,” while trihydroxylation of the B-ring could decrease “coarseness” [25]. A positive relationship is observed between polymer size, galloylation and astringency intensity in grape seeds. Symoneaux *et al.* [27] reported that the higher the tannin concentration, the more bitterness and astringency in wines. The increase in the DP (the degree of polymerization) is attributed to more astringent products, and the effect is reinforced by the concentration. Concerning bitterness, the DP appears to be less important, but a stronger sensation is observed for pentamers at a high concentration of procyanidins. Additionally, a positive correlation was found between the DP, percentage of galloylation and astringency intensity in apple seeds. A negative correlation was found between the percentage of prodelphinidins and the bitterness intensity in the skin.

### 2.3. The Relationship between Polysaccharides, Acids and the Intensity of Astringency in Fruit

Polysaccharide families clearly oppose the perception of astringency according to the results of a principal component analysis (PCAs), with the effect being stronger for mannoproteins and rhamnogalacturonan-II (RG-II). However, only polysaccharides rich in arabinose and galactose (PRAGs) were considered in a final fitted multiple linear regression (MLR) model, which explained 96.8% of the variability observed in the data. Oligosaccharides do not show clear opposition, revealing that the structure and size of carbohydrates are important for astringency perception. Mannose and galactose residues in the oligosaccharide fraction are positively related to astringency perception, most likely because their presence is due to the degradation of polysaccharides [28]. Several *in vitro* studies have shown that complex polysaccharides can disrupt protein-tannin interactions through different mechanisms, such as via inhibiting protein-tannin interactions [29,30] or inhibiting the precipitation of the protein-tannin complexes [31,32], causing polysaccharides to limit the concentration of available proanthocyanidins, thereby reducing astringency. Additionally, several polysaccharide families have been described as compounds that can interact with tannins [33,34] or with proanthocyanidin aggregates to yield soluble complexes [34]. Furthermore, the above-mentioned sensory studies performed with model wine revealed that acidic polysaccharides have a greater impact on the reduction of astringency perception. RG-II is the main acidic polysaccharide found in wines [35], and isolated fractions of this polysaccharide were shown to cause a significant decrease in overall astringency in the model solution, which was attributed mainly to changes in mouth lubrication and the formation of complexes with astringent compounds. Neutral polysaccharides also tend to decrease the intensity of astringency attributes. Nevertheless, the differences between the model wine and the fraction containing a mixture of mannoproteins and type II arabinogalactan proteins isolated from wine were not statistically significant [36].

Taking into account that all of the glycosyl residues found in the oligosaccharide fraction are also found in the polysaccharide fraction, it appears that the ability of carbohydrates to smooth out the perception of astringency is related to the size and tridimensional structure of the compounds. The mannose and galactose concentrations in the oligosaccharide fraction are positively related to astringency perception. This finding could be related to a decrease in the levels of mannoproteins and PRAGs and may not represent a direct effect of these glycoside residues on astringency perception. Commercial enzymatic preparations could play a role in the degradation of polysaccharides, leading to less protective oligosaccharides. A regression model constructed including the compositional variables and the perceived astringency allowed the authors to explain 96.8% of the variability observed in the data and to recognize the variables that were positively and negatively related to astringency perception [37].

In grape skins, tannins can interact with proteins and cell wall polysaccharides. The mechanism of tannin-protein binding involves hydrogen bonding and hydrophobic interactions [19]. Cell wall polysaccharides also contain hydroxyl groups and glycosidic oxygen atoms that have the ability to form hydrogen bonds and exhibit hydrophobic interactions with tannins [19,38]. Moreover, polysaccharides can also interact with tannins through covalent bonds [39].

Except for polysaccharides, the organic acids also have effect on astringency. Early in the last century, salivary volume, pH and protein composition were reported to change the flow rate, leding to the difference of the perceived intensity and duration of bitterness and astringency [40,41]. Guinard *et al.* pointed out that the astringency of low to moderate tannin-wine is effected by acidity adjustments [42]. Hanna *et al.* reported that astringency of alum was lowed equivalently by addition of equi-sour levels of lactic acid, citric acid or hydrochloric acid [43]. Increasing pH of cranberry juice contributed to low the intensity of astringency regardless of temperature or viscosity [44]. Coinciding with an increase in sourness, astringency decreased between pH 3.4 and 2.6 [45]. Beta-LG samples were more astringent than phosphate buffers, indicating that astringency was not caused by acid alone and that proteins contribute to astringency [46]. The astringency perception was lowered as ethanol level and pH values increased [47].

### 2.4. The Localization of Tannins in Fruit

In grapes, tannins are mainly located in the seeds and stems but are also found in the skins, where they are reactive and easily extractible. Tannins accumulate during early developing stages then decrease continuously in grape skins [48,49,50]. The phenolic compounds found in peanuts are mainly located in the skins and hulls [51], and although the weight of the skin is low in relation to the total nut, the skin contains a large proportion of the total polyphenols present in the nut. Nepote *et al.* [52] reported that the content of phenolic compounds in peanut skin was 115–149 mg/g of dry skin, depending on the solvent used. Yu *et al.* [53] found that the total phenolic content was approximately 90–125 mg/g of dry skin in peanut. In the grape berry, tannins are located in the seeds and skins, but their content and structure differ according to the location of the tissues. Seed proanthocyanidins contain only (epi) catechin subunits forming procyanidins [54], while skin proanthocyanidins also include (epi) gallocatechin subunits, forming prodelphinidins as well [55]. Peanut seed skin contains polyphenols with strong a-amylase inhibitory activity, which retards the absorption of carbohydrates and mainly functions by inhibiting a-amylase [56]. Plavac mali skin extracts show higher concentrations of anthocyanins and tannins, which results in a slightly higher astringency and lower intensity of bitterness perception [57]. Peng-Min L *et al.* [58] reported that tannins are the main phenolic compounds in astringent persimmons, and tannin concentrations are higher in pulp than in peel, thereby accounting for the greater phenolics concentration and antioxidant capacity in pulp. Tannin concentrations are very low in non-stringent persimmons, and their antioxidant capacity is mainly determined by other phenolics. Also, peel of apple [59], pear [60], peach [61], mango [62], pomegranate [63] and quince [64] contains more phenolics than pulp.

## 3. The Mechanism of the Biosynthesis of Astringent Substances

The biosynthetic pathways of condensed tannins and other phenolic compounds have been verified in a variety of crops. Tannins are generated through the following three pathways: the shikimic acid pathway (for shikimic acid), the phenylpropanoid pathway and the flavonoid pathway. The anthocyanins and condensed tannins in flavonoid compounds are the main components responsible for pigment and astringency in fruits, and can be measured easily. Assessment of the route of synthesis has been the main model approach for studying associated gene expression and the regulation of plant secondary metabolism.

**Figure 1:**
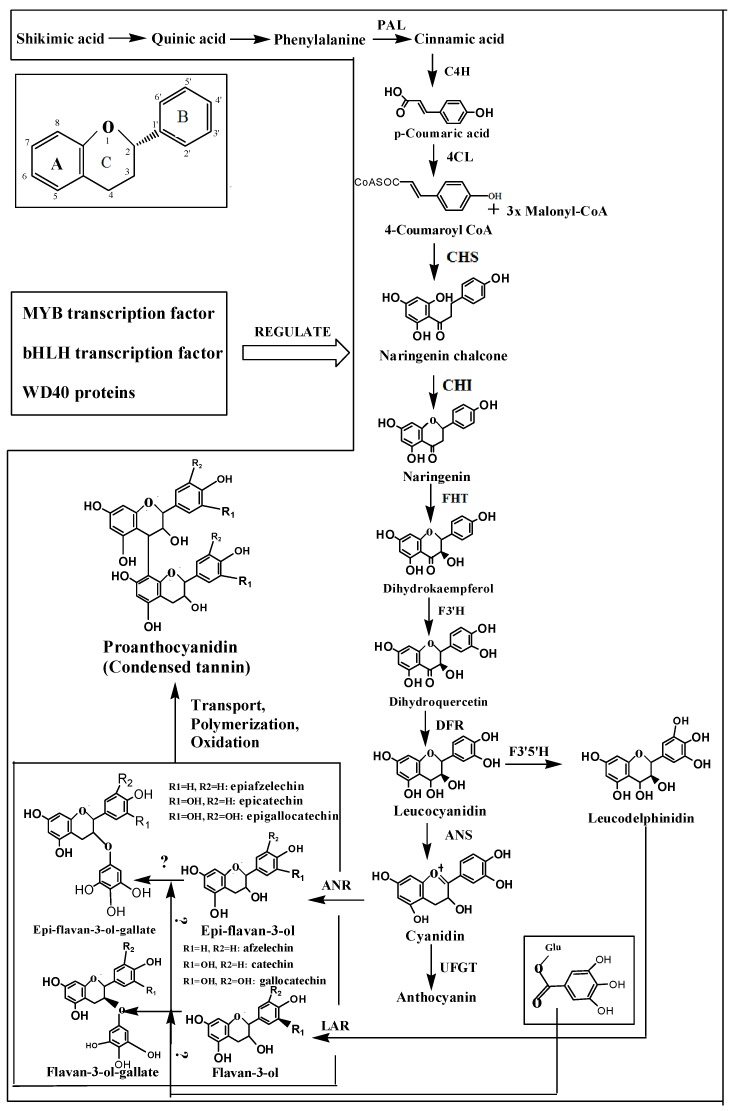
Tannin biosynthesis pathway.

PAL: Phenylalanine ammonia-lyase; C4H: Cinnamate 4-hydroxylase; 4CL: 4-coumaroyl CoA ligase; CHS: Chalcone synthase; CHL: Chalcone isomerase; F3'H: Flavanone 3-hydroxylase; DFR: Dihydroflavonol reductase; F3'5'H: Flavonoid 3'-5'-hydroxylase; LAR: Leucoanthocyanidin reductase; ANS: Anthocyanidin synthase; ANR: Anthocyanidin reductase; UFGT: Uridine diphosphate glucose-flavonoid glucosyltransferasev; MYB: Myeloblastosis; bHLH: basic helix-loop-helix.

### 3.1. Phenylalanine Ammonia-Lyase

PAL represents the first step in primary and secondary metabolic pathways, carrying out the first step in the reaction of catalyzing phenylalanine (Figure 1). The conversion of phenylalanine to cinnamic acid and ammonia via non-oxidative deamination plays a key role in the synthesis of flavonoids. Therefore, it has been the focus of many previous studies on plant secondary metabolism. *PAL* is a multi-gene family, and these genes control different metabolic pathways in different tissues.

### 3.2. Cinnamate 4-Hydroxylase

The catalysis reaction of phenylalanine with C4H is the second step in the pathway (Figure 1), but it is also the first oxidation reaction and involves the hydroxylation of cinnamic acid at its *para-*position. *trans-*Cinnamic acid is catalyzed to 4-hydroxycinnamic acid (*para*-position-coumaric acid). C4H is a member of the cytochrome P450 monooxygenase superfamily. An enzyme that requires oxygen and depends on NADPH to function properly was first discovered in pea shoots by Russell and Conn [65]. Thus far, the sequences of *C4H*s of a variety of crops have been obtained, and the expression of *C4Hs* is closely related to the lignification of plants.

### 3.3. Flavanone Hydroxylase

Flavanones are catalyzed by flavanone hydroxylase to produce dihydroflavonol (Figure 1). Flavanone hydroxylase is a key enzyme in the metabolic pathways of flavanones, protocatechuic acid, procyanidins and anthocyanin [66]. In reactions catalyzed by the F3H, F3'H and F3'5'H hydroxylases, flavanones are hydroxylated to generate several types of anthocyanin and tannin intermediates on the C-ring and at the 3' and 5' positions of the B-ring. F3'H and F3'5'H determine the extent and location of the hydroxylation of the B-ring in flavonoids, thereby affecting the structural stability and antioxidant function of flavonoids. Currently, research is mainly focused on the role of flavanone hydroxylase in the regulation of fruit and flower color. Flavanone hydroxylase has been extracted from many crops and is related to changes in fruit color. However, little is known about the role of flavanone hydroxylase in the tannin biosynthesis pathway.

### 3.4. Dihydroflavonol Reductase

Dihydroflavonol reductase (DFR) belongs to an NADPH-dependent short-chain DFR superfamily. It is a single gene encoding a key enzyme in the biosynthesis of tannins (Figure 1). The amino acid sequence of DFR determines the species of the substrate. The binding region of DFR in different species and substrates is highly conserved. The first 134 amino acid residues directly determine substrate specificity and are therefore divided into Asn-type DFR, Asp-type DFR and Asn/Asp-type DFR. Asn-type DFR occurs widely in plants, although there is only one form in monocotyledonous plants, whereas Asp-type DFR only exists in some dicots. In addition, only a few plants contain non-Asn/Asp-type DFR. Petit *et al.* described the three-dimensional structure of DFR in detail for the first time, in addition to studying the expression of the grape *DFR* in *E. coli* [67], separating and purifying the active protein, detecting DFR activity, and discovering the three-dimensional structure of the enzyme and the two conjugates of DHQ (DFR-DHQ and DFR-NADP) and coenzyme II (NADP). Trabelsi *et al.* further obtained crystals by conjugating both DFR-NADP-myricetin and quercetin DFR-NADP and identified flavonols that might inhibit the activity of DFR based on analysis of three-dimensional structure [68].

*DFR* was first isolated in 1985, from corn (*Zea mays*) and snapdragon (*Antirrhinum majus*), through transposon tagging [69]. Subsequently, Beld *et al.* isolated the petunia (Petunia hybrida) *DFR* [70] using a partial phenotypic *DFR* mutant gene from *Antirrhinum majus* as a probe. Employing homologous cloning methods, thus far, *DFRs* have been cloned from *Arabidopsis* (*Arabidopsis thaliana*), orchids (*Bromheadia finlaysoniana*), camellia (*Camellia sinensis*), tomato (*Lycopersicon esculentum*), rice (*Oryza sativa*) and other plants. Aida transferred *DFRs* to the ear of a blue pig using the Agrobacterium-mediated gene transfection method in 2000 [71]. Lo Piero *et al.* firstly reported the expression of *DFR* of fruit flesh *in vitro* whose biochemical features might be very different from the ones of *DFR* of flowers or leaves as well as non-producing anthocyanin species [72]. In recent years, the construction of expression vectors for *DFRs* has been completed for many crops, thus providing the basis for further regulation of the content of tannins. Previous studies have shown that changes in light, temperature and other external factors could affect the expression of *DFRs* [73].

### 3.5. Leucoanthocyanidin Reductase

The colorless anthocyanidin reductase gene belongs to the isoflavone reductase subfamily. It is a key structural gene involved in catalyzing the synthesis of condensed tannins (Figure 1). Flavan-3-trans-alcohol is formed under the function of colorless anthocyanin, also known as catechin. Tannin is the polymer of flavan-3-trans-alcohol. The *LAR* has been cloned from apples, grapes, legume and other plants, and the enzyme encoded by *LAR* catalyzes colorless anthocyanin to form flavan-3-alcohol [74,75]. In addition, studies have indicated that LAR constitutes the rate-limiting step in the DFR enzymatic reaction [76].

Different members of the *LAR* family play different roles in the flavonoid biosynthesis pathway and play space- and time-specific roles in different developmental stages. Colorless grape anthocyanin reductase genes regulate the type and accumulation of proanthocyanidins through their organization and spatial and temporal expression specificity [75]. The changes in the type and accumulation of flavonoids observed in strawberry fruit are closely related to *LAR* [77], and the highest expression of the *LAR* is observed during the early stage of the fruit development and maturation periods in apple, while the lowest expression is observed during the middle period [78,79]. The expression of *LARs* cloned from persimmon decreased gradually with maturing, but the content of condensed tannins increased in maturing persimmon [80].

### 3.6. Anthocyanin Synthase

Anthocyanin synthase (ANS), also referred to as anthocyanins dioxygenase (LDOX), belongs to the non-heme iron oxidase superfamily. It catalyzes leucocyanidin into colored anthocyanins (Figure 1), also known as epicatechins, which are transformed into cis-flavan 3-alcohol by anthocyanidin reductase and then generate condensed tannins [81].

### 3.7. Anthocyanin Reductase

Anthocyanidin reductase (ANR), encoded by the anthocyanin reductase gene *BAN* in *Arabidopsis* and *Medicago truncatula*, was first reported in 2003. ANR may catalyze anthocyanins into epicatechins through coordination with NADPH in plants [82] (Figure 1). *ANR* has been cloned from grapes [83], tea [84], oranges, apples, pears and other plants [74]. Most plants contain 1 to 2 *ANRs*. There are five introns in the *ANRs*, and their location and number are conserved. Bogs *et al.* reported that *ANR* can be expressed in the developing seeds, peel, flowers and leaves of grape, but the expression levels are different in different tissues. Overexpression of *ANR* in *Arabidopsis thaliana* is related to the content of tannins. Compared with *LAR*, the expression level of *ANR* is lower or insignificant in various colors of apple skin [75].

### 3.8. Function of the Main Enzymes are Described in Detail

PAL is closely related to flavonoid synthesis in strawberries [85], apples [86], grapes [87] and pears [88]. During fruit development, PAL activity shows two peaks: one in the young fruit and the other in mature fruit. It has been indicated that the divergent C4H isoforms contribute to the production of secondary metabolites [89]. C4H and 4CL play important roles in flavone biosynthesis and the efficiency of metabolic engineering in promoting flavone biosynthesis in *S. baicalensis* hairy roots [90]. F3H catalyses are involved in many biological activities including coloration of flowers, seeds and other plant organs, seed dormancy and longevity, ultraviolet radiation protection, antimicrobial activity, antioxidant activity, plant defense response to a broad spectrum of abiotic and biotic stress factors and medicinal properties [91,92,93]. The regulation of DFR have been studied in various plants [94,95,96,97] and the induction of DFR activity has been linked to an increase in condensed tannins accumulation, which may be important for defense against herbivores [98]. DFR can catalyze the reduction of three dihydroflavonols, kaempferol (DHK), dihydroquercetin (DHQ) and dihydromyricetin (DHM), into leucoanthocyanidins, which are common precursors for anthocyanin and condensed tannin synthesis [94]. It is different from that ANR and LAR are responsible for the production of (-)-epicatechin and (+)-catechin, respectively [74,75].

## 4. Regulation of the Content of Astringent Substances in Fruit

Tannins and catechins are key factors determining fruit astringency, seriously affecting the quality of fruit flavor. Regulating the concentrations of these substances to improve fruit quality is an effective strategy. At present, the methods for regulating astringent substances mainly include altering factors in the extracellular environment, hormonal levels related to intercellular signaling or intracellular gene regulation as well as RNA interference technology. Many studies have shown that the biosynthesis and accumulation of tannins are regulated by temperature [99], light [100], moisture [101] and other environmental conditions. Changes in environmental conditions will ead to changes in the content or structure of tannins and anthocyanins in fruit. These changes are achieved through modulating the expression of structural genes involved in the process of tannin synthesis. Cold stress can induce the expression of flavonoid genes in blood oranges and result in an increase of flavonoid levels [99]. Proanthocyanidins are generated by visible light, while ultraviolet light contributes to the synthesis of anthocyanins after young grape fruit have been exposed to visible and ultraviolet light [100].

Growth regulators play an important role in the process of the synthesis of astringent plant substances. Moalem-Beno *et al* found that the content of catechins in avocado calluses rises under the function of thidiazuron (TDZ) with cytokinin activity. The activity of F3H and DFR and catechin levels can be increased by spraying TDZ before harvesting [102]. Auxin, kinetin, chlormequat, abscisic acid and ethylene can induce the expression of *PALs* in plants.

A transcription factor is a protein that combines with specific DNA sequences within eukaryotic promoters or a protein with a DNA binding domain. It can interact with specific cis-acting elements in the promoter region, and through interactions with other related proteins, can activate or inhibit transcription. Astringent substances have been found to be involved in the synthesis of regulatory factors, including the *MYB* transcription factor, *MYC* family *bHLH* transcription factors and WD40 proteins.

Nesi *et al.* reported that *MYB* is a large gene family. The transcription factors of this family are involved in different types of plant secondary metabolism (such as that of anthocyanins and tannins) as well as hormone and stress responses, cell differentiation, the cell cycle and organ morphogenesis. In *Arabidopsis thaliana*, the *MYB* transcription factor participates in the metabolic regulation of tannin, and the expression of the major associated structurally regulated genes, including *DFR*, *ANS* and *ANR* [103]. *VvMYB5a*, *VvMYB5b*, *VvMYBPA1*, *VvMYBPA2* and many other *MYB* transcription factors have been isolated from grapes. *VvMYB5a* is expressed mainly during the early development of the peel, pulp and seeds. Heterologous expression in tobacco revealed that the synthesis of anthocyanins, flavonols, tannins and lignins is influenced by *VvMYB5a*, indicating that *VvMYB5a* can regulate different branches of the phenylalanine metabolism pathway. *VvMYB5b* is mainly involved in the flavonoid pathway, and increases in the content of anthocyanins and tannins are caused by the overexpression of this gene in tobacco. The metabolism of tannins in grapes is specifically regulated by *VvMYBPA1* and *VvMYBPA2*, which can activate the promoters of *LAR*, *ANR* and many structural genes in the flavonoid pathway. However, *VvMYBPA1* and *VvMYBPA2* do not bind to the *UFGT* promoter during the synthesis of celadon anthocyanin [104]. Five *MYB* transcription factors have been isolated from persimmon, in which the expression of *DkMyb4* and the expression patterns of *DkF3'5'H* and *DkANR* are very similar. The synthesis of tannins in persimmon is specifically regulated by *DkMyb4*. Heterologous expression in kiwifruit only causes the accumulation of tannins, without the accumulation of anthocyanins. Heterologous expression in kiwifruit also causes significant accumulation of tannins in persimmon calluses [105]. Furthermore, *DkMyb2* is involved in the metabolism of tannins in persimmon but does not cause continuous accumulation of tannins [106].

The *bHLH* transcription factors also belong to a multigene family. Different subfamilies play different roles in the growth and development, stress responses, signal transduction and regulation of the secondary metabolism of plants. *bHLHs* have been reported to participate in flavonoid metabolism or to be involved in the metabolism of tannins by controlling anthocyanin metabolism. The accumulation of anthocyanins in the peel and seeds is regulated by *VvMYCl* in grape. *VvMYCl* cannot activate the *CHI*, *UFGT* or *ANR* gene promoter by itself, but when it is co-transfected with *MYB* transcription factors, *VvMYCl* can significantly activate these three structural gene promoters [107].

Compared with the *MYB* and *bHLH* transcription factors, there have been few studies conducted on WD40 in plants. The scope of the WD40 family of proteins is quite extensive and selective. These proteins can interact with different proteins in different physiological and biochemical processes. WDR1 and WDR2 proteins have been isolated from grapes and show heterologous overexpression in wild-type Arabidopsis. There is no difference in the accumulation of anthocyanins between VvWDR2-transgenic plants and control plants, while WDR1-transgenic plants exhibit high accumulation of anthocyanin in the stems and leaves [108].

RNA interference technology can be used to further test and verify the function of genes. For example, the expression of *F3H* was significantly inhibited in soybeans using the RNA interference technique, and the content of flavonoids in the transgenic plant was shown to be significantly increased. Additionally, when walnut was reverse transformed with the chalcone synthase gene (*CHS*) by Eluch, it resulted in a decrease in the accumulation of flavonoids in the transgenic plants. RNA interference technology will undoubtedly play an important role in the regulation of the content of astringent substances in fruits.

## 5. Prospects for Studying Astringency of Fruit

The synthesis of astringent substances is controlled by a variety of structural and regulatory genes. The study of structural and regulatory genes is not only beneficial for clarifying the mechanism underlying the development and regulation of astringency in fruit at the molecular levels but also provides an effective means for the genetic improvement and metabolic engineering of fruit astringency regulation. At present, there are still some problems including the fact that (a) in most crops, the astringent substances are tannins, but the genes that control catechins and epicatechins to generate tannins in the astringent substance metabolism pathway are still not clear; and (b) the expression of transcription factor genes can activate the coordinated expression of multiple genes in specific branches of metabolism, but the types and quantities of transcription factors related to the formation of astringency are not the same in different crops. Rapid progress has been made in the study of the genomics, proteomics, transcriptomics and metabolisms of plant crops, which will contribute technologically to solving the above problems. The cloning and functional identification of genes in the astringency metabolic pathway and their spatio-temporal expression patterns as well as tannin biosynthesis-related transcription factor genes must be considered in future work to finally make it possible to control fruit astringent substances quantitatively.
